# A Reverse Genetics System for Cypovirus Based on a Bacmid Expressing T7 RNA Polymerase

**DOI:** 10.3390/v11040314

**Published:** 2019-04-01

**Authors:** Gaobo Zhang, Jian Yang, Fujun Qin, Congrui Xu, Jia Wang, Chengfeng Lei, Jia Hu, Xiulian Sun

**Affiliations:** 1 Wuhan Institute of Virology, Chinese Academy of Sciences, Wuhan 430071, China; zhanggb@wh.iov.cn (G.Z.); yangjian@wh.iov.cn (J.Y.); qinfj@wh.iov.cn (F.Q.); xucr@wh.iov.cn (C.X.); wangjia@wh.iov.cn (J.W.); cflei@wh.iov.cn (C.L.); hujia@wh.iov.cn (J.H.); 2 University of Chinese Academy of Sciences, Beijing 100049, China

**Keywords:** DpCPV, reverse genetics system, *vp80*-knockout, foreign protein

## Abstract

*Dendrolimus punctatus* cypovirus (DpCPV), belonging to the genus *Cypovirus* within the family *Reoviridae*, is considered the most destructive pest of pine forests worldwide. DpCPV has a genome consisting of 10 linear double-stranded RNA segments. To establish a reverse genetics system, we cloned cDNAs encoding the 10 genomic segments of DpCPV into three reverse genetics vectors in which each segment was transcribed under the control of a T7 RNA polymerase promoter and terminator tagged with a hepatitis delta virus ribozyme sequence. We also constructed a *vp80*-knockout *Autographa californica* multiple nucleopolyhedrovirus bacmid to express a T7 RNA polymerase codon-optimized for Sf9 cells. Following transfection of Sf9 cells with the three vectors and the bacmid, occlusion bodies (OBs) with the typical morphology of cypovirus polyhedra were observed by optical microscopy. The rescue system was verified by incorporation of a *Hin*dIII restriction enzyme site null mutant of the 9th genomic segment. Furthermore, when we co-transfected Sf9 cells with the reverse genetics vectors, the bacmid, and an additional vector bearing an *egfp* gene flanked with the 5′ and 3′ untranslated regions of the 10th genomic segment, aggregated green fluorescence co-localizing with the OBs was observed. The rescued OBs were able to infect *Spodopetra exigua* larvae, although their infectivity was significantly lower than that of wild-type DpCPV. This reverse genetics system for DpCPV could be used to explore viral replication and pathogenesis and to facilitate the development of novel bio-insecticides and expression systems for exogenous proteins.

## 1. Introduction

Cypoviruses (CPVs) belong to the family *Reoviridae* (genus *Cypovirus*) and typically have genomes comprising 10–11 double-stranded RNA (dsRNA) segments [1,2]. The icosahedral virions are embedded within a characteristic crystalline occlusion body (OB), which is formed by the polyhedrin protein encoded by the viral genome [3,4]. In the highly alkaline environment of the midgut, the OBs are cleaved to release virions which can infect midgut epithelial cells, resulting in delayed growth and even death of the infected larvae. During the period of illness and disease, the virus is continuously excreted in the feces and infects other healthy insects. CPV can also be passed on to the next generation of insects, contributing to a viral epidemic in the pest population [5]. Dendrolimus punctatus cypovirus (DpCPV) is a pathogen of *D. punctatus* and is considered the most destructive pest of pine forests worldwide [6]. DpCPV can infect 35 insect species spanning 10 *Lepidoptera* families [6]. This species has been applied in commercial insecticides to control the pine caterpillar, *D. punctatus*, since 1970 in Japan [7] and since 2010 in China [8].

DpCPV has a genome consisting of 10 segments of linear double-stranded RNA, designated from genomic segment 1 (S1) to S10 in decreasing order of size. In agreement with the nomenclature of Antheraea mylitta cypovirus (AmCPV) and Bombyx mori cypovirus (BmCPV), the proteins encoded by DpCPV S1 to S10 are called VP1, RdRp, VP2, VP3, NSP1, VP4, VP5, NSP2, NSP3 and polyhedrin, respectively [9,10,11,12,13].

Reverse genetics technology is a useful tool for studying different aspects of viral biology. Although reverse genetics systems exist for nearly all major groups of RNA viruses, including coxsackievirus [14], bunyavirus [15], coronavirus [16], zika virus [17], and respiratory syncytial virus [18], it is more challenging to rescue infectious CPV from cDNAs because of the technical complexity of manipulating its 10 genomic segments and the inability of virions to spread among the available cell lines.

Previously, a temperature-sensitive reovirus strain was rescued using an RNA-based reverse genetics system and rotaviruses bearing engineered changes in the viral attachment proteins were isolated using a partially plasmid-based reverse genetics system. Only one or two rotavirus genomic fragments could be replaced with other viral genes from different species [19]. In 2007, Kobayashi et al. developed an entirely plasmid-based reverse genetics system for animal double-stranded RNA viruses by co-transfecting murine L929 fibroblast cells with 10 plasmids encoding gene segments of mammalian orthoreovirus cDNAs flanked by the T7 RNA promoter and hepatitis delta virus ribozyme sequence (HDV Rib) and a recombinant vaccinia virus expressing T7 polymerase [20]. Several years later, an improved reverse genetics system for mammalian orthoreoviruses was developed by cloning the cDNAs encoding multiple reovirus genome segments into a few plasmids, reducing the number of recombinant vectors required from 10 to 4 and improving the efficiency of virus recovery [21]. Subsequently, other reoviruses, such as rotavirus [22] and bluetongue virus [23], were successfully recovered using a similar approach. Recently, it has been demonstrated that rotavirus can be rescued by overexpression of some viral genes without the need for a helper plasmid [24]. An RNA-based reverse genetics system for BmCPV was developed by co-transfection of cultured BmN cells with in vitro-transcribed S1–S10 RNA segments [25].

In this report, we describe the establishment of a plasmid-based reverse genetics system for DpCPV. Infectious DpCPV virions can be rescued by co-transfection of Sf9 cells with three plasmids encoding cDNAs derived from 10 DpCPV genomic segments and a *vp80*-knockout AcMNPV bacmid expressing T7 RNA polymerase. Using this system, we rescued a recombinant DpCPV containing an exogenous enhanced green fluorescent protein (*egfp*) gene.

## 2. Materials and Methods

### 2.1. Viruses, Plasmids, Bacterial Strains, Cells, and Insects

DpCPV (hereinafter called DpCPV-WT) was initially isolated from *D. punctatus* larvae during a natural outbreak [10]. The pFastBac-Dual donor plasmid (pFD) (Invitrogen, Carlsbad, CA, USA), pMD19-T Simple plasmid (Takara, Tokyo, Japan), pBlueScript plasmid (pBS) (Invitrogen), HST08 competent cells (Takara), DH10B cells containing an AcMNPV bMON14272 bacmid (AcBac) and a pMON7124 helper plasmid (Invitrogen) were used for the experiments. The pFD-VP39-mCherry plasmid was constructed previously in our laboratory [26]. DH10B cells (Invitrogen) containing a *vp80*-knockout barmaid were kindly provided by Dr. Zhihong Hu, Wuhan Institute of Virology (WIV), Chinese Academy of Sciences, Wuhan, China. *Spodoptera frugiperda* cells (Sf9) were maintained at 27 °C in Grace’s medium (Invitrogen) supplemented with 10% fetal bovine serum (FBS) (Gibco, Grand Island, NY, USA) and 0.1% (*v*/*v*) antibiotic-antimycotic solution (Invitrogen). *S. exigua* larvae were provided by the core facility and technical support, WIV. The larvae were reared on artificial diets at 28 °C.

### 2.2. Construction and Identification of Recombinant Bacmids as A Source of T7 Polymerase

The T7 RNA polymerase segments were codon-optimized for Sf9 cells and synthesized at Sangon Biotech (Shanghai, China) Co., Ltd. The codon-optimized T7 polymerase segments were PCR-amplified using the forward primer F-*Hin*dIII-T7pol and the reverse primer R-*Bam*HI-T7pol (sequences listed in Table 1) by KOD–Plus-Neo (Toyobo, Osaka, Japan). The resulting amplicons were inserted into the pFastBac-Dual plasmid using the *Hin*dIII and *Bam*HI restrictions sites under the control of the *polyhedrin* promoter to generate pFD-P_PH_-T7pol.

**Figure 1:**
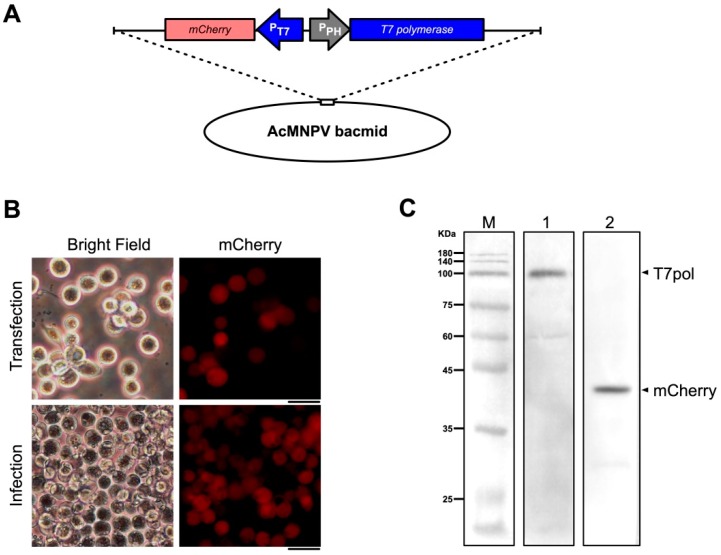
Construction and identification of an AcMNPV bacmid (AcBac-P_PH_-T7pol-P_T7_-mCherry) containing genes encoding T7 polymerase and mCherry. (**A**) Schematic representation of AcBac-P_PH_-T7pol-P_T7_-mCherry. The codon-optimized *T7 polymerase* sequence was incorporated downstream of the *polyhedrin* promoter and the *mCherry* gene downstream of the *T7* promoter (P_T7_). (**B**) Sf9 cells were transfected with AcBac-P_PH_-T7pol-P_T7_-mCherry and infected with vAcBac-P_PH_-T7pol-P_T7_-mCherry. At 96 h post-transfection, the supernatants were harvested and used to infect a new culture of Sf9 cells. Images were obtained at 72 h post-transfection and post-infection by fluorescence microscopy with 561-nm light excitation. Scale bar, 20 μm. (**C**) Western blotting analyses of Sf9 cells infected with vAcBac-P_PH_-T7pol-P_T7_-mCherry. Lanes: M, molecular size marker; 1, primary antibody: mouse anti-T7 polymerase (1:2000 dilution); 2, primary antibody: mouse anti-mCherry antibody (1:2000 dilution). The bands were indicated with arrows and names.

To generate AcBac-P_PH_-T7pol-P_T7_-mCherry, the donor plasmid pFD-P_PH_-T7pol-P_T7_-mCherry was first constructed (Figure 1A). The linear pFD-P_PH_-T7pol segments were PCR-amplified with primers F-pFD-delp10 annealing upstream of the *p10* promoter and R-pFD-delp10 (Table 1) annealing at the HSV TK poly(A) signal to delete the *p10* promoter and the multiple cloning site. The T7pro-mCherry segments were PCR-amplified from the pFD-VP39-mCherry plasmid using primers F-T7pro-mCherry and R-mCherry (Table 1) bearing 16-bp homologous fragments of pFD-P_PH_-T7pol at both ends. The pFD-P_PH_-T7pol-P_T7_-mCherry vector was obtained by transforming competent *Escherichia coli* HST08 cells with a mixture of the vector and insert using the FastCloning method [27]. Briefly, the vector and insert were PCR-amplified with 16-base terminal overlapping sequences. After digesting the mixture of amplified vector and insert with *Dpn*I to eliminate the DNA template used in the PCR reaction, competent cells were directly transformed with the mixture. The AcBac-P_PH_-T7pol-P_T7_-mCherry was obtained by transforming DH10B competent cells harboring the pMON7124 helper plasmid and the AcBacmid bMON14272 with the pFD-P_PH_-T7pol-P_T7_-*m*C*herry* plasmid via the Bac-to-Bac^®^ Baculovirus Expression System (Invitrogen). The bacterial cells were cultured (37 °C, 225 rpm, 4 h), after which they were serially diluted 10-fold (10^−1^, 10^−2^, and 10^−3^) in SOC medium (0.5% yeast extract, 2% tryptone, 10 mM NaCl, 2.5 mM KCl, 10 mM MgCl_2_, 10 mM MgSO_4_, 20 mM glucose), and 50 μL of each dilution was plated onto an LB agar plate containing 50 μg/mL kanamycin, 7 μg/mL gentamicin, 10 μg/mL tetracycline, 100 μg/mL X-gal, and 40 μg/mL IPTG. White colonies were picked from the plate, re-streaked on fresh LB agar plates, and incubated for 48 h at 37 °C. The plates were then incubated overnight at 37 °C. Next, a single clone was picked, inoculated in liquid culture, and grown overnight. The resulting AcBac-P_PH_-T7pol-P_T7_-mCherry bacmid was isolated according to the Bac-to-Bac^®^ Baculovirus Expression System protocol (Invitrogen).

When the Sf9 cell density reached 30–40%, the cells were transfected with 8 μg of AcBac-P_PH_-T7pol-P_T7_-mCherry using Cellfectin™ II Reagent (Thermo Scientific, Waltham, MA, USA) following standard procedures. At 96 h post-transfection, the supernatants were harvested and used to infect a new culture of Sf9 cells.

To generate AcBac-T7pol-Δvp80, DH10B competent cells harboring the pMON7124 helper plasmid and AcBac-Δvp80 were transformed with the donor plasmid pFD-P_PH_-T7pol via the Bac-to-Bac system.

### 2.3. Construction of Three Recombinant Plasmids Bearing Full-Length cDNAs of DpCPV S1–S10 dsRNAs and An Additional Plasmid, T-S10UTR-egfp

The sequence of HDV Rib (GenBank accession number DD147565.1, 1 to 85 nt) linked to a T7 RNA polymerase terminator (GenBank accession number KJ782405.1, 5203 to 5331 nt) was synthesized at Sangon Biotech (Shanghai) Co., Ltd. and cloned into the pMD19-T simple vector. The resulting vector was called T-HDV Rib. To construct T-T7pro-S1~S10, the DpCPV S1–S10 segments were PCR-amplified from DpCPV cDNAs [28] and cloned into the T-HDV Rib vector (Figure S1A). To construct T-T7pro-S9M, DNA segments were PCR-amplified from the T-T7pro-S9 vector with specific primers F-DpCPV-S9M and R-DpCPV-S9M (Table 1) containing 16-bp homologous fragments and self-ligated using the FastCloning method [27] (Figure S1B).

To construct pBS-S6-S5-S3, the DpCPV S6, S5 and S3 segments containing T7 promoters and the HDV Rib sequences were sequentially cloned into pBS vector using the FastCloning method. The pBS-S8-S4-S2 and pBS-S10-S7-S1-S9 vectors were constructed using the same methods (Figure S1C). The pBS-S10-S7-S1-S9M vector was constructed by ligating the S9M segments from T-T7pro-S9M and pBS-S10-S7-S1 segments using a method similar to that shown in Figure S1A.

To construct T-S10UTR-egfp, the linear T-S10UTR vector with a deleted open reading frame (ORF) was PCR-amplified from T-T7pro-S10. The *egfp* gene segments amplified from the pEGFP-N1 plasmid (Clontech, Heidelberg, Germany) were ligated into T-T7pro-S10 using the methods described above (Figure S1D).

All recombinant vectors were confirmed by PCR and DNA sequencing at Sangon Biotech (Shanghai) Co., Ltd.

### 2.4. Rescue of DpCPV OBs in Sf9 Cells

When the Sf9 cell density reached 30–40%, Sf9 cells (about 9 × 10^5^ cells per well in a 6-well tissue culture plate) were transfected with mixtures of the DNA vectors constructed above and AcBac-T7pol-Δvp80 (2 μg of each vector and 8 μg of bacmid) using Cellfectin™ II Reagent (Thermo Scientific) and standard procedures. To rescue rDpCPV, the vectors included in the DNA mixture were pBS-S6-S5-S3, pBS-S8-S4-S2 and pBS-S10-S7-S1-S9. To rescue rDpCPV-S9M, the vectors included in the DNA mixture were pBS-S6-S5-S3, pBS-S8-S4-S2 and pBS-S10-S7-S1-S9M. To rescue rDpCPV-egfp, the DNA mixture contained an additional T-S10UTR-egfp vector. The transfected cells were cultured in Grace’s Insect Medium (Invitrogen) supplemented with 10% FBS. Seven days post-transfection, rDpCPV and rDpCPV-S9M OBs were observed under inverted optical microscopy and rDpCPV-*egfp* OBs were observed under a fluorescence microscope with 488-nm light excitation. The cells were frozen and thawed three times, and 1.5 mL of virion-containing cells from each culture plate were collected and transferred to a sterile 2-mL tube as the P1 viral stock.

To obtain large amounts of recombinant viruses, third-instar *S. exigua* larvae were fed with an artificial diet including virus-containing cells and reared at 28 °C before they were dissected. DpCPV OBs were collected from the *S. exigua* midgut by differential centrifugation (Briefly, 300× *g* for 5 min to remove debris, 3000× *g* centrifugation for 30 min to collect the precipitate, repeated three times) as the P2 viral stock. The P3 viral stocks were obtained from the midguts of *S. exigua* infected with DpCPVs from the P2 viral stocks.

### 2.5. Identification of Rescued DpCPVs

Genomic dsRNAs were extracted from OBs to confirm recombinant viruses from the P3 viral stocks. Briefly, 500 μL of 0.2 mol/L Na_2_CO_3_-NaHCO_3_, pH 10.8, were added to 1 mL of purified OB suspension (10^6^ OBs/mL). After the mixture turned clear, the pH was adjusted to 7.4 with 1 mol/L Tris-HCl, pH 6.8, and centrifuged at 3800× *g* for 5 min to remove debris. The supernatant was treated with 15 μL of proteinase K (50 mg/mL) at 50 °C for 15 min [29]. The dsRNAs were extracted using TRIzol reagent (Invitrogen), analyzed on 1% agarose gels and stored at −70 °C. Prior to reverse transcription, the RNA samples were fully digested with RNase-free DNase I to avoid genomic DNA contamination. The HiScript^®^ 1st strand cDNA synthesis kit (Invitrogen) was used to synthesize cDNAs from the genomic dsRNA segments.

DpCPV S9 genomic segments were PCR-amplified from the cDNAs of DpCPV variants using the F-T7pro-S9 and R-S9-HDV primers (Table 1). The purified PCR products were digested with *Hin*dIII at 37 °C to identify differences between rescued DpCPV variants and DpCPV-WT.

### 2.6. Insecticidal Bioassays

The droplet-feeding method was used to determine the infectivity of DpCPV-WT and recombinant DpCPVs [30]. Briefly, third-instar *S. exigua* larvae were starved overnight and fed an artificial mixture of 4% sucrose, 10% erioglaucine disodium salt (Aladdin industrial corporation, Shanghai, China), and different concentrations (1 × 10^5^–3 × 10^8^ OBs/mL) of CPVs. Specifically, the final concentrations of DpCPV-WT were 1 × 10^5^, 3 × 10^5^, 1 × 10^6,^ 3 × 10^6^ and 1 × 10^7^ OBs/mL. The final concentrations of rDpCPV were 3 × 10^6^, 1 × 10^7^, 3 × 10^7^ and 1 × 10^8^ OBs/mL; those of DpCPV-S9M were 1 × 10^6^, 3 × 10^6^, 1 × 10^7^, 3 × 10^7^ and 1 × 10^8^ OBs/mL; and those of rDpCPV-egfp were 3 × 10^6^, 1 × 10^7^, 3 × 10^7^, 1 × 10^8^ and 3 × 10^8^ OBs/mL. After feeding for 10 min, larvae with blue guts were picked to a new 24-well plate containing the artificial diet. Forty-eight larvae were used for each viral treatment. Larvae fed on the artificial diet and treated with only distilled water were used as a control. The larvae were kept at 27 °C and 60–70% humidity. Larval mortality was recorded daily until all the tested larvae had either died or pupated. The LC_50_ and 95% confidence interval (CI) for each virus were determined by probit analysis using SPSS 13.0 (SPSS Inc., Chicago, IL, USA) [31]. The LC_50_ values of rDpCPV, rDpCPV-S9M and rDpCPV-egfp were compared to DpCPV-WT using a potency ratio [32].

### 2.7. Reverse Transcription-Quantitative PCR (RT-qPCR)

The F-qPCR-egfp and R-qPCR-egfp primers (Table 1) were used to amplify the *egfp* gene segment (720 bp) and construct the T-qPCR-egfp vector to make a standard curve [33]. Copy number of the T-qPCR-egfp vector was calculated using the *SciencePrime* web server (http://scienceprimer.com/copy-number-calculator-for-realtime-pcr). The probe primer Probe-qPCR-egfp (Table 1) was flanked with 5′-FAM and 3′BHQ1 moieties. Genomic dsRNA segments were extracted from 1 × 10^6^ OBs of different viruses and diluted into 40 μL of RNase-free water. The cDNAs were transcribed from dsRNA segments as the template for RT-qPCR using HiScript®II 1st Strand cDNA Synthesis Kit (Vazyme, Nanjing, Jiangsu, China). The RT-qPCR was performed on a Bio-Rad CFX96^TM^ Real-Time PCR System (Bio-rad, Berkeley, CA, USA) using the AceQ qPCR Probe Master Mix kit (Vazyme). According to the manufacturer’s protocol, the thermal cycling program was as follows: 1 cycle at 95 °C for 5 min, 40 cycles at 95 °C for 10 s, 60 °C for 30 s. The amplification results were expressed as the threshold cycle number (Ct). Copy number of the *egfp* gene was calculated using the ΔΔCt method based on the standard. The primers used for RT-qPCR are displayed in Table 1.

The copy numbers of the DpCPV-S2, S9 and S10 genes were calculated by a similar method using T-T7pro-S2, and T-T7pro-S9 and T-T7pro-S10 as standards, respectively. Differences among the copy numbers of a gene from different viruses were analyzed by one-way ANOVA with a post-hoc Tukey’s multiple comparison if the *F* value was significant.

### 2.8. Scanning Electron Microscopy (SEM) and Transmission Electron Microscopy (TEM)

For SEM, the OBs were purified from *S. exigua* larvae according to the method described above and resuspended in phosphate-buffered saline (PBS, 137 mM NaCl, 2.7 mM KCl, 10 mM Na_2_HPO_4_, and 2 mM KH_2_PO_4_, pH 7.4) at 4 °C prior to analysis. The OBs were spread onto a piece of foil paper, dried naturally at room temperature overnight, sputter-coated with gold and observed with a scanning electron microscope (SU8010, Hitachi, Tokyo, Japan) at an accelerating voltage of 10 kV.

For TEM, OBs purified from the midguts of *S. exigua* larvae were stored in 40% (*v*/*v*) glycerol at 4 °C prior to analysis. The OBs were then embedded in resin, sectioned, and stained as previously described [34]. The stained ultrathin sections were examined with a Hitachi H-800 transmission electron microscope (Hitachi Co., Ltd., Tokyo, Japan) at an accelerating voltage of 200 kV. The solid and empty virions embedded in OBs were counted for DpCPV-WT (7 OBs), rDpCPV (10 OBs), rDpCPV-S9M (14 OBs), and rDpCPV-egfp (10 OBs). The ratios of solid and empty virions were compared by the *χ*^2^ test using SPSS 13.0.

### 2.9. Western Blotting

At 7 days post-transfection with AcBac-T7pol-Δvp80 or 5 days post-infection with vAcBac-P_PH_-T7pol-P_T7_-mCherry, Sf9 cells were blown down, centrifuged and resuspended in 32 μL of PBS. The cell debris was lysed in 10 μL of sample buffer (5 × SDS-PAGE buffer) in a boiling water bath for 10 min and loaded onto 12% bisacrylamide gels containing 4% stacking gels along with a prestained protein ladder (Thermo Scientific). After electrophoresis for 90 min, the proteins were transferred to polyvinylidene difluoride membranes (Millipore, Billerica, MA, USA) for 60 min at 100 V. The membranes were blocked overnight in Tris-buffered saline (TBS) containing 5% (*w*/*v*) nonfat dry milk at 4 °C and then washed three times in TBS-T buffer solution (50 mmol/L Tris-HCl, 200 mmol/L NaCl, 0.1% Tween 20, pH 7.5) for 15 min with agitation. The membranes were incubated with primary antibody (i.e., mouse anti-T7 polymerase, 1:2000 dilution, Novagen, Billerica, MA, USA; anti-mCherry antibody, 1:2000 dilution, Beyotime, Nantong, China) to identify the expression of T7 polymerase and mCherry for 3 h at 37 °C and washed three times for 10 min with TBS-T. The membranes were probed with goat anti-mouse secondary antibody (1:2000 dilution) conjugated to horseradish peroxidase (Boster, Wuhan, China). Bound antibody was visualized using the Genesnap gel imaging system (SynGene, Cambridge, England).

## 3. Results

### 3.1. A Codon-Optimized T7 RNA Polymerase Can be Expressed in Sf9 Cells

T7 RNA polymerase was codon-optimized for Sf9 cells (GenBank accession number MK567984) and synthesized. An AcMNPV bacmid, AcBac-P_PH_-T7pol-P_T7_-mCherry, in which the T7 polymerase was under the control of the *p10* promoter and mCherry was under the control of the T7 promoter, was constructed using the Bac-to-Bac system (Figure 1A). Red fluorescence was observed in Sf9 cells by fluorescence microscopy at 48 h post-transfection with this bacmid (Figure 1B), and the expression of T7 polymerase and mCherry proteins was confirmed by western blotting (Figure 1C). These results indicated that the codon-optimized T7 RNA polymerase could be expressed in Sf9 cells and that the T7 promoter initiated the expression of a downstream foreign gene.

**Figure 2:**
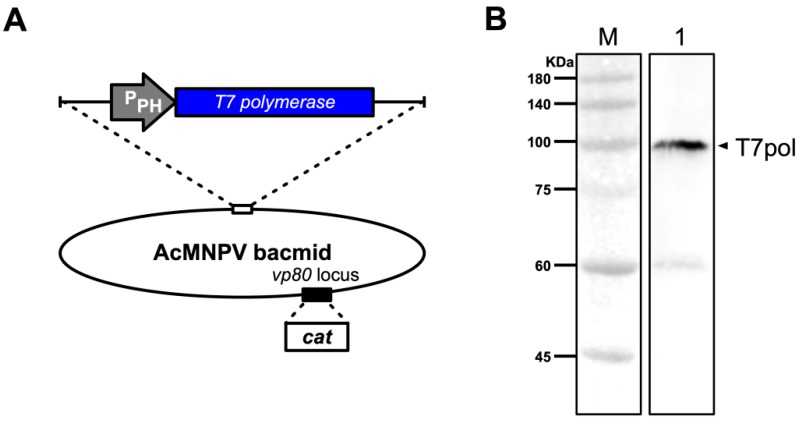
Construction and identification of a AcMNPV bacmid (AcBac-T7pol-Δvp80) expressing T7 polymerase. (**A**) Schematic representation of AcBac-T7pol-Δvp80. The codon-optimized *T7 polymerase* ORF was incorporated downstream of the *polyhedrin* promoter (P_PH_) of the *vp80*-knockout AcMNPV bacmid. (**B**) Western blotting analyses of Sf9 cells transfected with AcBac-T7pol-Δvp80. Lanes: M, molecular size marker; 1, primary antibody: mouse anti-T7 polymerase (1:2000 dilution).

It has been reported that the recombinant AcMNPV bacmid lacking the *vp80* ORF (AcBac-Δvp80) does not affect expression of genes downstream from the *ph* and *p10* promoters, as it does not produce infectious virions following transfection of Sf9 cells [35]. Thus, the T7 polymerase was inserted downstream of the *polyhedrin* promoter (P_PH_) of AcBac-Δvp80 to obtain the bacmid AcBac-T7pol-Δvp80 as a source of T7 RNA polymerase (Figure 2A). Western blotting indicated that T7 polymerase was expressed after transfection of Sf9 cells with this bacmid (Figure 2B).

### 3.2. OBs Could be Recovered by Co-Transfection of T7 RNA Polymerase-Expressing Bacmid and Three Plasmids Containing cDNAs Derived from the 10 dsRNA Segments of DpCPV

**Figure 3:**
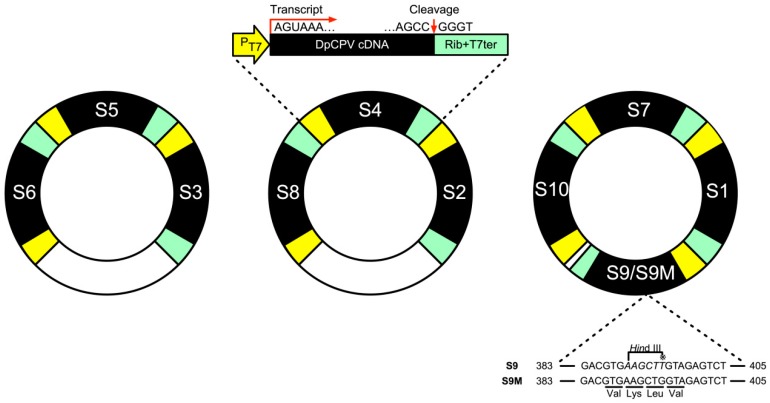
Schematic representation of the reverse genetics vectors containing cDNAs derived from *Dendrolimus punctatus* cypovirus (DpCPV) RNA segments. Three or four gene transcription cassettes encoding DpCPV cDNAs flanked by the T7 RNA polymerase promoter and an HDV Rib and T7 RNA polymerase terminator cassette were combined into single plasmids, creating three constructs for the reverse genetics systems of rDpCPV or rDpCPV-S9M (see Figure S1C). The single nucleotide difference in S9 unique to rDpCPV-S9M and DpCPV is shown in the alignment as an asterisk. The T/G substitution at position 395 was a deliberate change engineered into the cloned DpCPV S9 cDNA fragment used as a marker for rescue.

DpCPV contains 10 dsRNA genomic segments, to reduce the number of plasmids required for reverse genetics, three P_T7_-DpCPV cDNAs (S3, S5 and S6, or S2, S4 and S8), or four P_T7_-DpCPV cDNAs (S1, S7, S9 and S10) containing HDV Rib and T7 RNA polymerase terminator cassettes were introduced into the rescue backbone plasmid pBS to produce thepBS-S6-S5-S3, pBS-S8-S4-S2 and pBS-S10-S7-S1-S9 vectors (Figure 3). To distinguish the rescued DpCPVs from DpCPV-WT, a *Hin*dIII restriction enzyme site null mutation was incorporated into the DpCPV S9 cDNA (G to T at nucleotide 395) to produce the pBS-S10-S7-S1-S9M vector (Figure 3).

**Figure 4:**
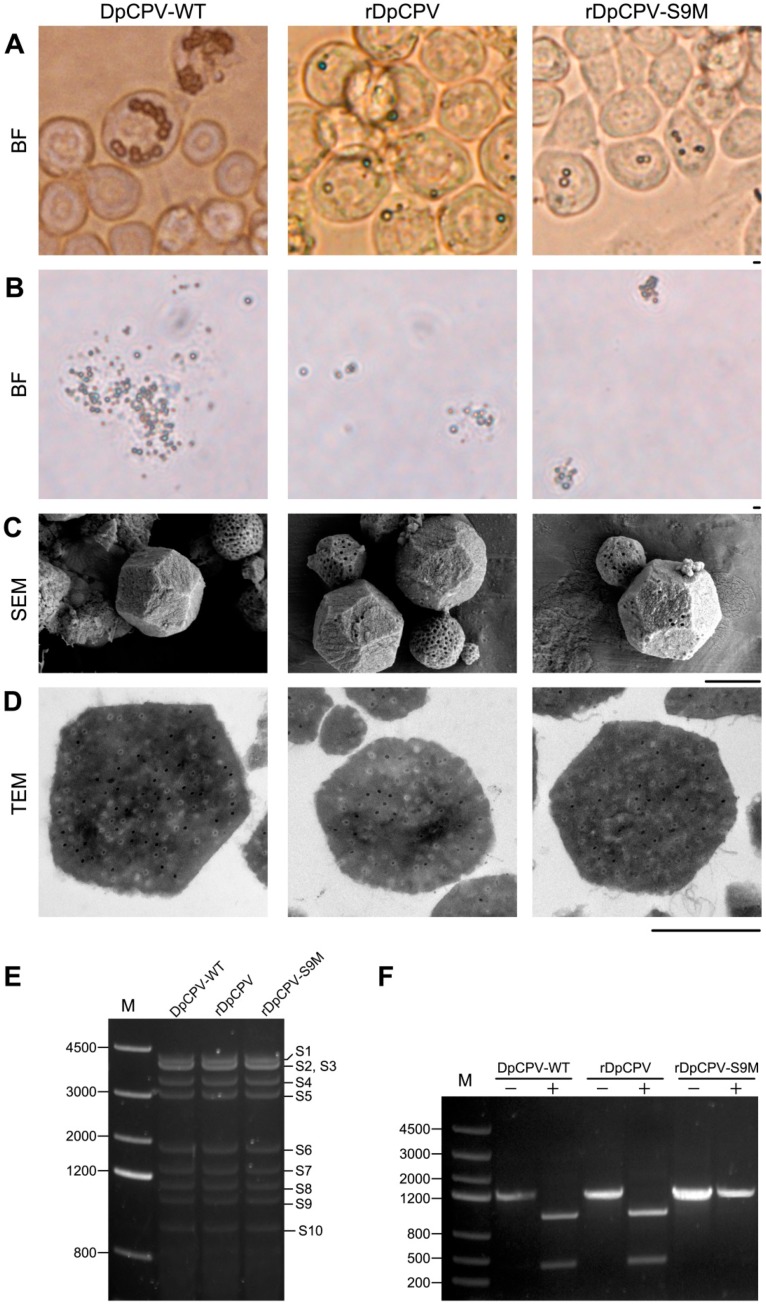
Generation and identification of the rescued DpCPVs. (**A**) OBs in Sf9 cells were obtained by transfection with the three DpCPV constructs shown in Figure 3 and AcBac-T7pol-Δvp80, and observed by optical microscopy. The Sf9 cells infected with DpCPV-WT were used as a control. Scale bars, 10 μm. To compare the morphology of wild-type and rescued DpCPVs, the OBs were purified from midguts of *S. exigua* larvae and diluted to 1 × 10^6^ OBs/mL and observed by optical microscopy (**B**), scanning electron microscopy (**C**) and electron microscopy (**D**). Scale bars, 1 μm. There was no significant morphological differences between the wild-type and rescued DpCPVs. (**E**) Agarose gel electrophoresis of the genomes of DpCPV-WT, rDpCPV, and rDpCPV-S9M. M, molecular size marker. (**F**) Reverse transcription PCR products derived from the S9 genome segment of DpCPV-WT and recombinant rDpCPV, rDpCPV-S9M and rDpCPV-egfp were analyzed by agarose gel electrophoresis before and after digestion with *Hin*dIII. −, undigested RT-PCR product; +, *Hin*dIII-digested RT-PCR product.

Sf9 cells were co-transfected with the three plasmids (pBS-S10-S7-S1-S9, pBS-S6-S5-S3 and pBS-S8-S4-S2) along with AcBac-T7pol-Δvp80. OBs with the typical morphology of cypovirus polyhedra were observed at 7–8 days post-transfection (rDpCPV) (Figure 4A). The site-directed mutant viruses (rDpCPV-S9M) were also rescued when the pBS-S10-S7-S1-S9 vector was replaced with pBS-S10-S7-S1-S9M (Figure 4A). Sf9 cells were also transfected with a mixture of the three DpCPV constructs (pBS-S10-S7-S1-S9, pBS-S6-S5-S3 and pBS-S8-S4-S2) or AcBac-T7pol-Δvp80 separately as controls, but no OBs could be observed.

### 3.3. Morphological Comparison and Detection of Genetic Markers Confirmed that DpCPVs were Correctly Rescued

Since DpCPV virions cannot spread among Sf9 cells, the number of OBs in the transfected cells were limited. Third-instar *S. exigua* larvae were fed on diets contaminated with the transfected cells to amplify the rescued viruses. The resulting OBs from the midguts of *S. exigua* larvae could be observed under an inverted optical microscope (Figure 4B), a scanning electron microscope (Figure 4C), and a transmission electron microscope (Figure 4D). The morphology of the rescued viruses was consistent with DpCPV-WT.

The RNA segments extracted from OBs of rDpCPV and rDpCPV-S9M were identical to those of DpCPV-WT by agarose gel electrophoresis (Figure 4E). The extracted RNA was used as a template to obtain cDNAs by reverse transcription, and the DNA fragments of S9 were amplified by PCR and digested with *Hin*dIII. The S9 DNA fragments of DpCPV-WT and rDpCPV were digested into two bands, while those of DpCPV-S9M could not be digested (Figure 4F).

### 3.4. Exogenous RNA Segments Might be Incorporated into CPV Particles

**Figure 5:**
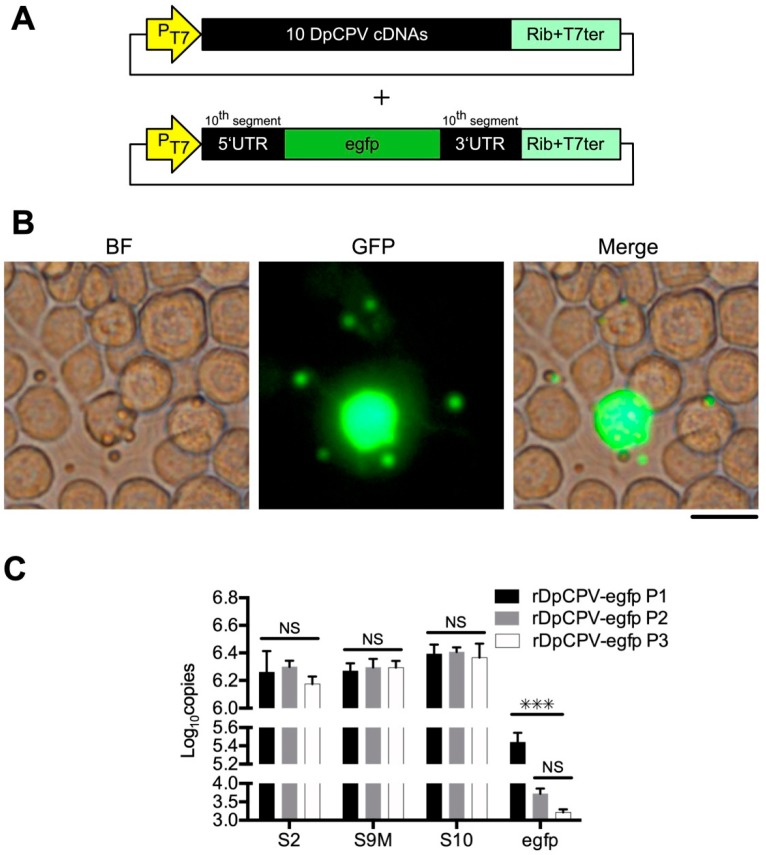
Generation and identification of DpCPV-egfp containing an *egfp* gene flanked with the 5′ and 3′ UTR of the 10th genomic segment. (**A**) Schematic representation of the T-S10UTR-egfp vector in which the ORF of the DpCPV S10 genome was replaced with the *egfp* gene. T-S10UTR-egfp was mixed with three DpCPV constructs which contained the 10 DpCPV cDNAs. (**B**) Sf9 cells were transfected with the three DpCPV constructs (pBS-S10-S7-S1-S9M, pBS-S6-S5-S3 and pBS-S8-S4-S2), AcBac-T7pol-Δvp80 and an extra vector T-S10UTR-egfp to generate rDpCPV-egfp. Images were obtained by fluorescence microscopy with 488-nm light excitation at 7 days post-transfection. OBs were observed, and some OBs co-localized with green fluorescence. Scale bars, 10 μm. (**C**) The rDpCPV-egfp successively infected *S. exigua* larvae for three generations and the copy numbers of the S2, S9, S10 and *egfp* genes in equivalent numbers of OBs were determined by RT-qPCR. With passaging, there was no significant change in the copy numbers of the S2, S9 and S10 genes, while the copy number of *egfp* in the P2 and P3 generations was significantly lower than in the P1 generation (NS indicates *p* > 0.05, *** indicates *p* < 0.001, Tukey’s multiple comparison post *F* test).

It has been reported that CPV genomic fragments are anchored at the apexes of 12 turrets inside the capsid [3]. To investigate if DpCPV could encapsulate exogenous RNA segments, a recombinant virus containing an exogenous *egfp* gene was rescued using the methods described above. The T-S10UTR-egfp DNA segment was constructed by replacing the ORF region of DpCPV S10 in the T-DpCPV-S10 vector with the *egfp* gene (Figure 5A). At 2 days post-co-transfection of Sf9 cells with T-S10UTR-egfp, three vectors (pBS-S10-S7-S1-S9M, pBS-S6-S5-S3 and pBS-S8-S4-S2) and AcBac-T7pol-Δvp80, green fluorescence was observed in some cells under a fluorescence microscope. OBs could be observed at 7 days post-transfection, and some OBs co-localized with green fluorescence (Figure 5B). After three passages in *S. exigua* larvae, the viruses purified from the midgut were called rDpCPV-egfp P1, rDpCPV-egfp P2 and rDpCPV-egfp P3, respectively. Though a large number of OBs were collected and their morphology was identical to DpCPV-WT, green fluorescence could not be observed under the fluorescence microscope, indicating EGFP was not incorporated into the OBs. The specific gene markers (modified at *Hin*dIII site of the S9) of rDpCPV-egfp were also confirmed via the methods above. The RNA bands isolated from the OBs were consistent with those of DpCPV-WT, while the cDNA of the S9M fragments could not be digested by *Hin*dIII.

After the genomic dsRNA segments were extracted from 10^6^ OBs of the DpCPV variants and reverse transcribed into cDNAs, the copy numbers of S2, S9 and S10 genes in equivalent numbers of OBs were determined by RT-qPCR. The copy numbers of the S2, S9 and S10 genes did not change significantly during the three passages (*F* = 1.119, *p* = 0.386, *F* = 2.02, *p* = 0.823 and *F* = 0.486, *p* = 0.673, respectively) (Figure 5C).

To determine the proportion of *egfp* RNA encapsulated into viral particles, the copy number of *egfp* in equivalent numbers of OBs during three serial passages of rDpCPV-egfp was determined using qPCR. With passaging, the copy number of *egfp* was significantly reduced (*F* = 57.782, *p* < 0.001) (Figure 5C). In the first passage of rDpCPV-egfp in *S. exigua* larvae, the ratio of the *egfp* copy number to that of S2 was 14.37%. After two successive passages, the ratio in the rDpCPV-egfp P2 and rDpCPV-egfp P3 virions decreased to 0.27% and 0.11%, respectively, indicating that the *egfp* gene could be encapsulated in the virus, but the ratio of foreign genetic material decreased rapidly with passaging.

### 3.5. Infectivity of the Rescued DpCPVs was Lower than that of DpCPV-WT

**Figure 6:**
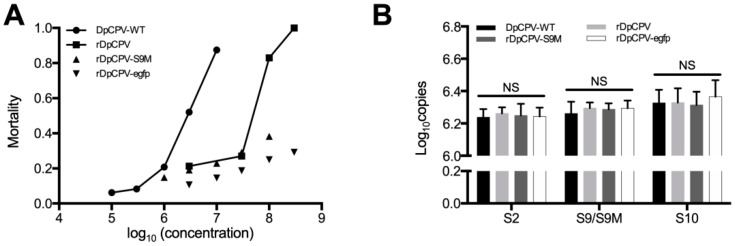
Comparison of virulence and gene copy numbers between DpCPV-WT and recombinant DpCPVs. (**A**) *S. exigua* larvae were infected with different concentrations of DpCPV-WT, rDpCPV, rDpCPV-S9M and rDpCPV-egfp. Mortality induced by viruses was related to the concentrations used for inoculation. The virulence of DpCPV-WT and rDpCPV was greater than that of rDpCPV-S9M and rDpCPV-egfp in which S9 was replaced with S9M. (**B**) The S2, S9 and S10 gene copy numbers in equivalent numbers of OBs of different DpCPVs were determined by RT-qPCR. There was no significant change in the copy numbers of the S2, S9 and S10 genes between DpCPV-WT and recombinant DpCPVs (NS indicates *p* > 0.05, *F* test).

The LC_50_ of rDpCPV was 25.7 × 10^6^ OBs/mL, significantly higher than that of DpCPV-WT (2.47 × 10^6^ OBs/mL) (Table 2). Although we increased the concentrations of rDpCPV-S9M and rDpCPV-egfp virus to 1 × 10^8^ and 3 × 10^8^ OBs/mL, respectively, the mortality of inoculated larvae was still less than 50% (Figure 6A).

### 3.6. Genetic Stability of the Rescued DpCPVs when Passaged in Host Insects

To compare the genetic stability of the rDpCPVs and DpCPV-WT, the copy numbers of the S2, S9 and S10 genes in equivalent numbers of OBs from the third passage of DpCPV-WT, rDpCPV, rDpCPV-S9M and rDpCPV-egfp were determined by qPCR. There was no significant difference in the copy numbers of the S2 (*F* = 0.109, *p* = 0.952), S9 (*F* = 0.238, *p* = 0.867) and S10 (*F* = 0.226, *p* = 0.876) genes between DpCPV-WT and the rescued viruses (Figure 6B), indicating that endogenous genomic segments were stably packaged in the viral particles.

Solid and empty DpCPV-WT, rDpCPV, rDpCPV-S9M and rDpCPV-egfp virions were counted by TEM observation. The ratios of solid to empty particles were not significantly different between DpCPV-WT and the rescued viruses (all *p* > 0.05, Pearson *χ*^2^ test) (Table 3), indicating that endogenous genomic segments were packaged in the virions in a certain ratio.

## 4. Discussion

Understanding of the molecular biology of dsRNA viruses is progressing slowly because RNA cannot be directly manipulated at the molecular level. This problem has been partially solved with the emergence of reverse genetics systems. Due to the technical complexity of the multi-segmental dsRNA genomes of reoviruses, the development of reverse genetics systems has been slower. Although an RNA-based BmCPV reverse genetic system has been established [25], several issues such as easy degradation of the single-stranded RNA obtained in vitro and inefficient transfection of excess fragments have not been fully resolved.

The main goal of our study was to develop a plasmid-based system for recovery of genetically modified DpCPV from cell cultures. Initially we tried to construct an insect cell line that stably expressed T7 polymerase, but due to the large size of the protein, we found that it could not be fully transcribed and expressed by several promoters (e.g., *OpIE-1* [36], *OpIE-2* [37], *Drosophila* metallothionein [38], *Densoviral* P9 [39]). Therefore, a helper virus like the recombinant vaccinia virus was adapted for expression of T7 polymerase. After transfection of Sf9 cells with AcBac-P_PH_-T7pol-P_T7_-mCherry and infection with helper virus, the red fluorescence indicated that T7 polymerase could be expressed under the *polyhedrin* promoter, indicating that it might recognize and initiate transcription of reporter genes in Sf9 cells (Figure 1B). In reverse genetics systems using recombinant vaccinia virus as a helper virus, the helper virus can be removed from the rescued virus by differential temperature sensitivity or cellular tropism. It has been reported that the *vp80*-knockout bacmid does not produce infectious baculoviruses, and does not affect expression of exogenous genes [34]. Thus, we utilized AcBac-T7pol-Δvp80 to express the T7 polymerase to diminish the effects of the baculovirus (Figure 2A). Using FastCloning technology, 10 DpCPV cDNA fragments were constructed in three vectors (Figure 3). DpCPVs were rescued by co-transfecting Sf9 cells with the vectors and AcBac-T7pol-Δvp80. By morphological comparison and detection of genetic markers, we have developed a robust and efficient reverse genetics system for DpCPV (Figure 5).

In several reverse genetics systems of RNA viruses, such as enterovirus 71 [40], respiratory syncytial virus [18] and Bombyx mori cypovirus [25], the virulence of rescued viruses was more or less lower than those of the wild-type viruses. In this system, the LC_50_s (95% CI) of DpCPV-WT and rDpCPV viruses were 2.47 (0.97–8.06) and 25.7 (7.6–75.3) × 10^6^ OBs/mL, which is in accord with the rescued RNA viruses mentioned above. In this study, more than 60% *S. exigua* larvae survived after treatment with rDpCPV-S9M (1 × 10^8^ OBs/mL) or rDpCPV-egfp (3 × 10^8^ OBs/mL). According to the RT-qPCR and TEM results, there was no significant difference in the copy numbers of viral nucleic acids (Figure 6B) and the ratio of solid particles (Table 3) among the rescued and wild-type viruses, indicating that rDpCPVs contain the same genomic sequences as the wild-type DpCPV, while its virulence is much lower than that of wild-type DpCPV. For rDpCPV and rDpCPV-S9M, the mutation of S9 to S9M in the ORF region did not affect genome assembly. Through RNA structure prediction, we found that the S9M caused a structural change of the RNA segment, which may have caused the decline in virulence. We speculate that this structural region may be involved in some viral processes, but the specific mechanisms need further study. Though the virulence of rDpCPV-S9M was lower than that of rDpCPV, the S9M null-mutation was used to confirm the rescue system worked correctly, did not contaminated by wild-type DpCPV.

The turret structures inside the capsid of CPV have been reported to be key sites for anchoring genomic fragments [3]. The DpCPV genome consists of 10 dsRNA segments, while the capsid contains 12 turrets, and thus we speculated that the capsid could accommodate more foreign genes. When T-S10UTR-egfp was added to the transfected mixture, co-localization of green fluorescence and OBs was observed in transfected Sf9 cells by fluorescence microscopy (Figure 5B). However, no green fluorescence was observed in OBs purified from the midguts of *S. exigua* larvae. The assembly of CPV particles may adapt a precise mechanism, thus it was not proposed that the exogenous EGFP might be incorporated into virions or OBs. We speculate that the *egfp* RNA was encapsulated in the virions as an extra fragment and that the gene could be expressed in Sf9 cells. The RT-qPCR results showed that some of the *egfp* genetic material was indeed encapsulated into rDpCPV-egfp particles, whereas not every rDpCPV virion encapsulated an additional gene. The ratio of the *egfp* to S2 gene RNA segment copy number in rDpCPV-P1 was 14.37%, indicating that virions containing *egfp* accounted for 14.37% of the total, and this ratio decreased significantly in successive passages. We speculate that this reduction was due to the lack of resistance screening during passages. There was no significant difference in the copy number of the S10 gene between rDpCPV from P1 to P3 (Figure 5C). Though the untranslated regions (UTRs) of T-S10UTR-egfp and S10 were identical, they were not competitively encapsulated in virions. The UTRs of rotaviruses have been shown to be key regions for recognition of viral genes packaged into virions [41]. When we replaced the ORF regions of S1–S10 with the *egfp* gene, we found that the predicted RNA structure of the UTR region did not change when the S2 and S10 ORF regions were replaced (Figure S2). When the ORF region of the S2 gene was replaced with the *egfp* gene, co-localization of OBs and green fluorescence could also be observed in cells. The plasmids where *egfp* replaced the ORF region of the other DpCPV fragments were constructed and performed similarly in co-transfections. However, although OBs could be observed for the S7 and S9 substitution, no co-localization of green fluorescence and OBs was observed (Figure S3).

As a commercial insecticide with a wide host range, DpCPV has a long infection cycle and low virulence. Its icosahedral structure may be used to display of foreign proteins [42], and we expect that its insecticidal activity may be increased by expressing foreign proteins on the capsid surface, such as fusion-associated small transmembrane protein [43] and enhancing factors [44]. This will be an important direction for our future research into improving the efficacy of encapsulating foreign genes into recombinant viruses.

In summary, we have successfully established a reverse genetics system for DpCPV by co-transfection of Sf9 cells with three reverse genetics vectors and a *vp80*-knockout bacmid as a source of T7 polymerase. Utilizing this system, we engineered infectious rDpCPVs carrying *egfp* genes with the UTRs of DpCPV S10. This highly efficient reverse genetics system and the resulting recombinant DpCPVs expressing exogenous genes will be useful tools for studying the molecular biology of DpCPV and developing next-generation biological insecticides and expression vectors.

## Figures and Tables

**Table 1:** Primers used in the study.

Primer	Sequence (5′–3′) ^a,b^
F-*Hin*dIII-T7pol	CCCAAGCTTATGAACACAATCAACATCGCTAAGAACG
R-*Bam*HI-T7pol	CGCGGATCCTTAAGCGAAGGCGAAGTCGGACTC
F-pFD-delp10	CGTATACTCCGGAATATTAATAGATC
R-pFD-delp10	CGGCAATAAAAAGACAGAATAAAACG
F-T7pro-mCherry	TATTCCGGAGTATACGTAATACGACTCACTATAGGGCGCC
R-mCherry	*TGTCTTTTTATTGCCG*CTTGTACAGCTCGTCCATGCCG
F-T-T7pro	*ATAGTGAGTCGTATTA*ATCTCTGGAAGATCCGCGCGTACC
R-T-HDV	GGGTCGGCATGGCATCTCCA
F-T7pro-S1	*TAATACGACTCACTATA*GAGTAAAGTGTATGTCTATACCAGAA
F-T7pro-S2	*TAATACGACTCACTATA*GAGTAAAAGTCAGTATCTTACCGGCA
F-T7pro-S3	*TAATACGACTCACTATA*GAGTAAAGACAGATGACGAGAGAC
F-T7pro-S4	*TAATACGACTCACTATA*GAGTAATTTCCACCATGTGGCATTATAC
F-T7pro-S5	*TAATACGACTCACTATA*GAGTAATTTCCCCGTCACTTAAAGG
F-T7pro-S6	*TAATACGACTCACTATA*GAGTAAGATTCCGCAATATCCCATGG
F-T7pro-S7	*TAATACGACTCACTATA*GAGTAATTTGGTCATAACAGCAAAG
F-T7pro-S8	*TAATACGACTCACTATA*GAGTAAAGTCCAGTACTAGTTAAAGAC
F-T7pro-S9	*TAATACGACTCACTATA*GAGGAAATCCCAGGTGTAAACCGAAT
F-T7pro-S10	*TAATACGACTCACTATA*GAGTAAAAGTCAGTATCTTACCGGCA
R-S1-HDV	*GAGATGCCATGCCGACCC*GGCTAACGGTCGTGTATGAATGAGG
R-S2-HDV	*GAGATGCCATGCCGACCC*GGCTAACTCTGAACAGCGTACATC
R-S3-HDV	*GAGATGCCATGCCGACCC*GGCTAACGGTCGACACATGTTCATGC
R-S4-HDV	*GAGATGCCATGCCGACCC*GGCTAACGTTTCCCACCCC
R-S5-HDV	*GAGATGCCATGCCGACCC*GGCTAACCATCTCCCCGTG
R-S6-HDV	*GAGATGCCATGCCGACCC*GGCTAACGTTGACTCCGCTT
R-S7-HDV	*GAGATGCCATGCCGACCC*GGCTAACGTTTGGTCACTCCG
R-S8-HDV	*GAGATGCCATGCCGACCC*GGCTAACGGTAGTCCAGCCTGTTG
R-S9-HDV	*GAGATGCCATGCCGACCC*GGCTAACTACCCAGTGCCCTAAGG
R-S10-HDV	*GAGATGCCATGCCGACCC*GGCTAACTGTCAGTCAGTACCGC
F-T7ter-pBS	*ATATCCGGATGGTACC*GTCATAGCTGTTTCCTGTG
R-pBS-T7pro	*ATAGTGAGTCGTATTA*CATGATTACGCCAAGCGC
R-T7ter	GGTACCATCCGGATATAGTTCCTCC
F-T7ter-pBS-2	*ATATCCGGATGGTACC*TTGCGCGCTTGGCGTAATC
R-pBS-T7pro-2	*ATAGTGAGTCGTATTA*TTAACCCTCACTAAAGGGAACAAAAG
F-T7ter-pBS-3	*ATATCCGGATGGTACC*GCTTTTGTTCCCTTTAGTGAGGG
R-pBS-T7pro-3	*ATAGTGAGTCGTATTA*TGGGTACCGGGCCCCCCC
F-T7ter-pBS-4	*ATATCCGGATGGTACC*CCGCTCTAGAACTAGTGGATCCC
R-pBS-T7pro-4	*ATAGTGAGTCGTATTA*GATCCACTAGTTCTAGAGCGGCCG
F-DpCPV-S9M	*AGCTGGTAGAGTCTAC*TCCCCTGC
R-DpCPV-S9M	*GTAGACTCTACCAGCT*TCACGTCGG
F-egfp	*TAATACGTAAAGGATC*ATGGTGAGCAAGGGCGAGG
R-egfp	*CAAGTTACACGAGCAA*TTACTTGTACAGCTCGTCCATGCC
F-T-S10	TTGCTCGTGTAACTTGGATACCAG
R-T-S10	GATCCTTTACGTATTATGCCGG
Probe-qPCR-S2 ^c^	GCTAGAAGTGGGAGGTGACGTAGCAGC
F-qPCR-S2	TGAGGCATGGCTAAATTTCC
R-qPCR-S2	AACCGCCTGCATAACAATTC
Probe-qPCR-S9 ^c^	TTACGCCCAGCGCATCTCACCC
F-qPCR-S9	TGGTATGGGTAAAATCAGGTCTTG
R-qPCR-S9	TCGAGGATGCGAAATTTACATATG
Probe-qPCR-S10 ^c^	ACTATCCTAATGGCGGCGACGCGCA
F-qPCR-S10	CAAGGAGTATCGCGAAGGGC
R-qPCR-S10	ATTTGGATCGCACGTGGCTT
Probe-qPCR-egfp ^c^	AGGCTACGTCCAGGAGCGCACCATCTT
F-qPCR-egfp	CCACATGAAGCAGCACGACT
R-qPCR-egfp	GGGTCTTGTAGTTGCCGTCG

^a^ Restriction sites are underlined; ^b^ Homologous fragments are indicated with italics; ^c^ The probe primers were flanked with 5′-FAM and 3′BHQ1 moieties.

**Table 2:** Infectivity of wild-type and rescued DpCPVs against third-instar *S. exigua* larvae.

Virus	LC_50_ (95% CI) (×10^6^ OBs/mL)	Potency Ratio (95% CI) to DpCPV-WT ^a^
DpCPV-WT	2.47 (0.97–8.06)	-
rDpCPV	25.7 (7.6–75.3)	11.4 (1.3–1586.5)
rDpCPV-S9M	>100	
rDpCPV-egfp	>300	

^a^ The potency ratio was calculated by dividing the LC_50_ of DpCPV-WT by that of the rescued virus. The significance of differences was based on whether the 95% confidence interval (CI) of the potency ratio included 1.0.

**Table 3:** Ratio of solid to empty virions in the OBs of wild-type and rescued DpCPVs.

Virus	Section Numbers	Solid Particles	Empty Particles	Ration	*χ* ^2^	*p*
DpCPV-WT	7	268	205	1.307		
rDpCPV	10	149	119	1.252	0.079	0.779
rDpCPV-S9M	14	206	167	1.233	0.174	0.677
rDpCPV-egfp	10	225	183	1.230	0.203	0.652

## Supplementary Materials

The following are available online at https://www.mdpi.com/1999-4915/11/4/314/s1, Figure S1: Flowchart for generation of reverse genetics vectors containing cDNAs of DpCPV RNA segments, Figure S2: Folding of the 5′ and 3′ UTRs of the DpCPV S1 to S10 genomic segments and S1UTR-egfp to S10UTR-egfp segments, Figure S3: Sf9 cells were transfected with the three DpCPV constructs (pBS-S10-S7-S1-S9M, pBS-S6-S5-S3 and pBS-S8-S4-S2), AcBac-T7pol-Δvp80 and one of the additional vectors, T-S1UTR-egfp to T-S9UTR-egfp.
