# Supplementary material for: A Reverse Genetics System for Cypovirus Based on a Bacmid Expressing T7 RNA Polymerase
Source: Viruses. 2019 Apr 1;11(4):314. doi: 10.3390/v11040314 (PMC6521135; doi:10.3390/v11040314)
Supplement: Supplementary file 1 [file viruses-11-00314-s001.pdf]

Supplementary Materials:

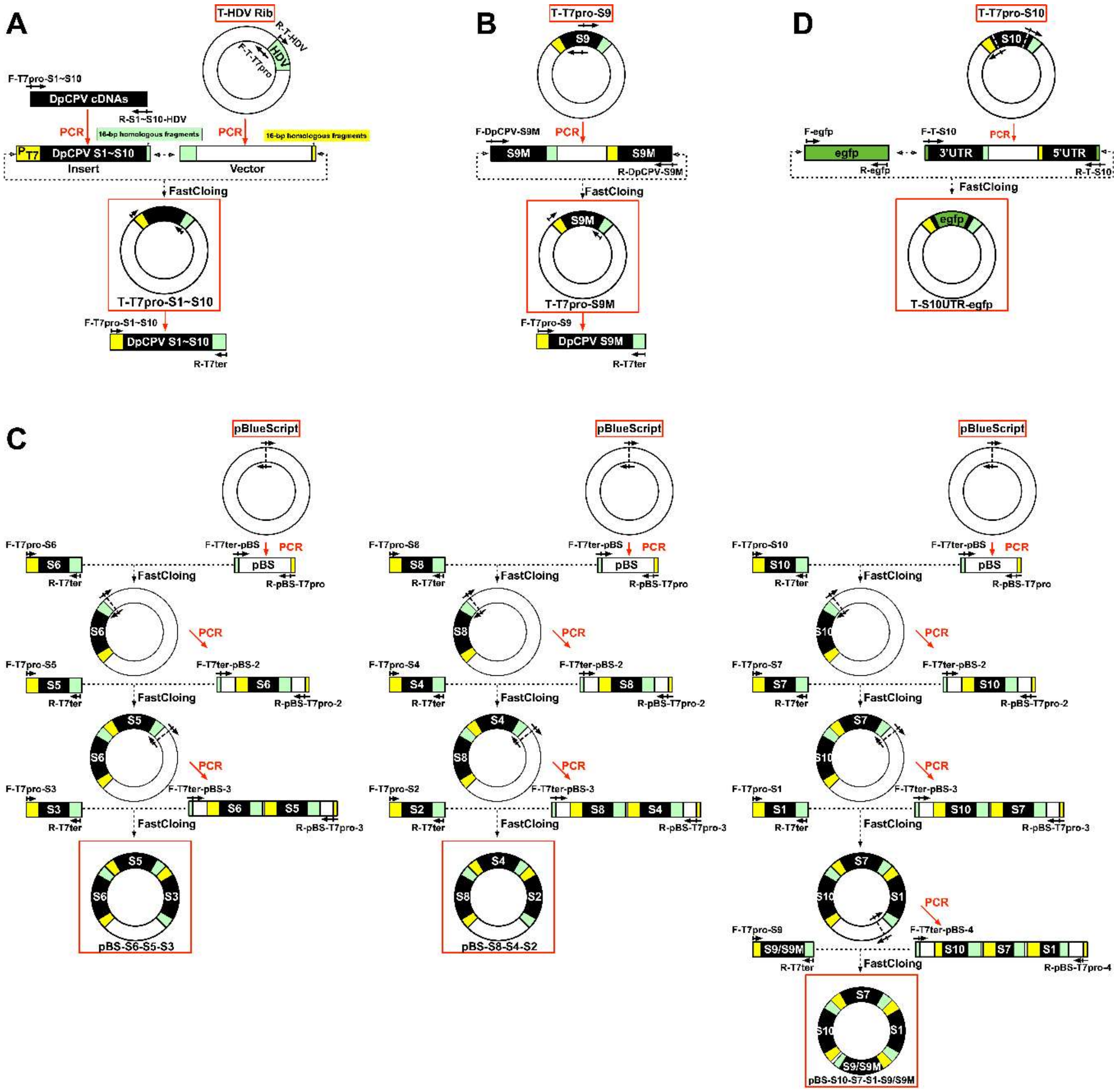

Figure S1. Flowchart for generation of reverse genetics vectors containing cDNAs of DpCPV RNA segments. The T-T7pro-S1~S10 (**A**) and T-T7pro-S9M (**B**) vectors were constructed as the templates for the reverse genetics vectors. (**C**) S6 was cloned into pBS to produce pBS-S6, S5 was cloned into pBS-S6 to produce pBS-S6-S5, and S3 was cloned into pBS-S6-S5 to produce pBS-S6-S5-S3. The pBS-S8-S4-S2 and pBS-S10-S7-S1-S9/S9M vectors were constructed by a similar process. (**D**) T-S10UTR-egfp was constructed as an additional vector for rDpCPV-egfp. The red arrows indicate PCR reactions and the black dotted arrows indicate FastCloning reactions. The black arrows indicate primers. The 16-bp homologous fragments at both ends of the amplified vector and insert were labeled with the same color.

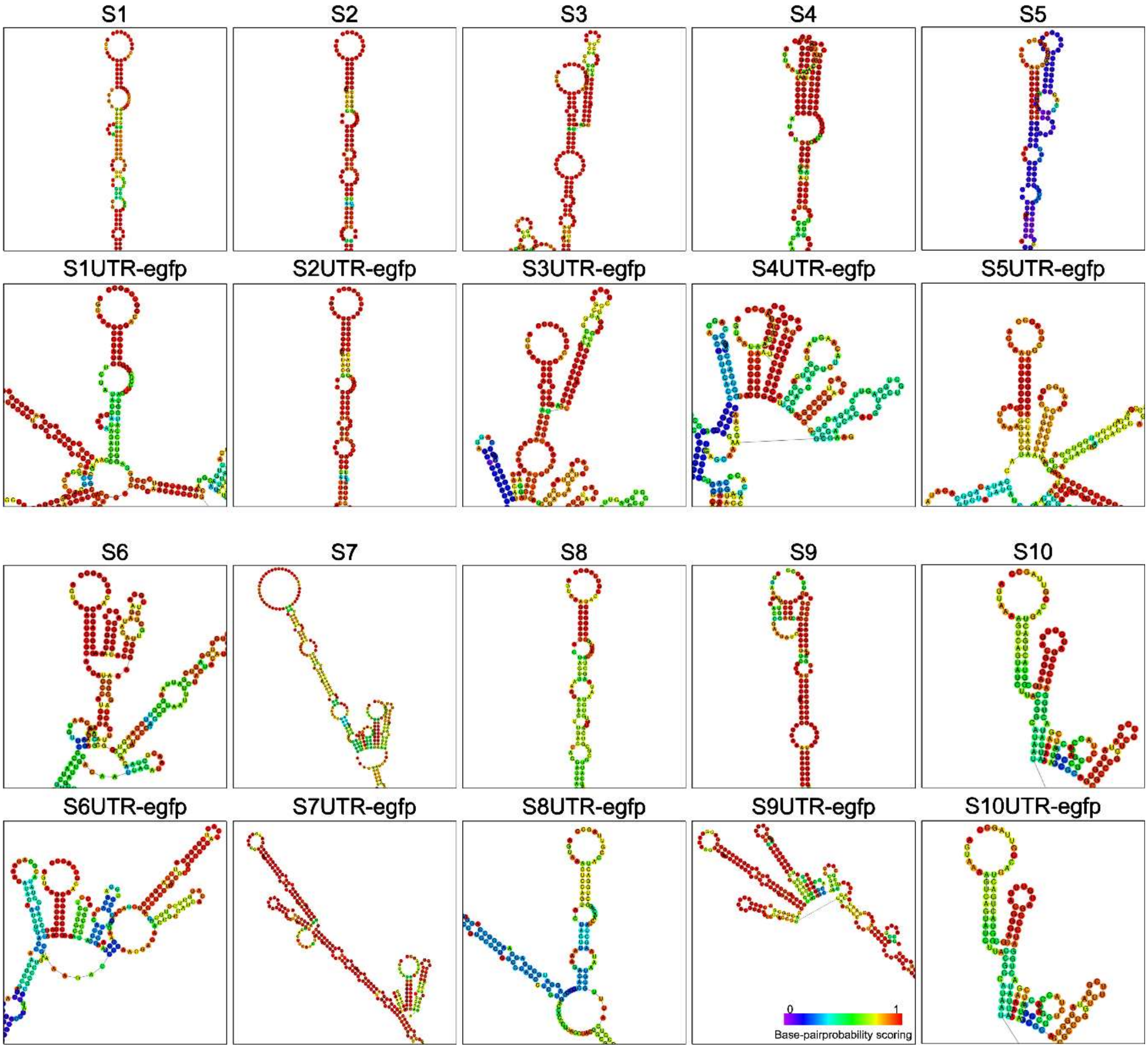

Figure S2. Folding of the 5′ and 3′ UTRs of the DpCPV S1 to S10 genomic segments and S1UTR-egfp to S10UTR-egfp segments. The *RNAfold* web server (http://rna.tbi.univie.ac.at/cgi-bin/RNAfold.cgi) was used to calculate the minimum free energy secondary structure and base pair probabilities for the +RNA of DpCPV S10 and S10UTR-egfp constructed by replacing the ORF regions in the S1 to S10 genes with the *egfp* gene.

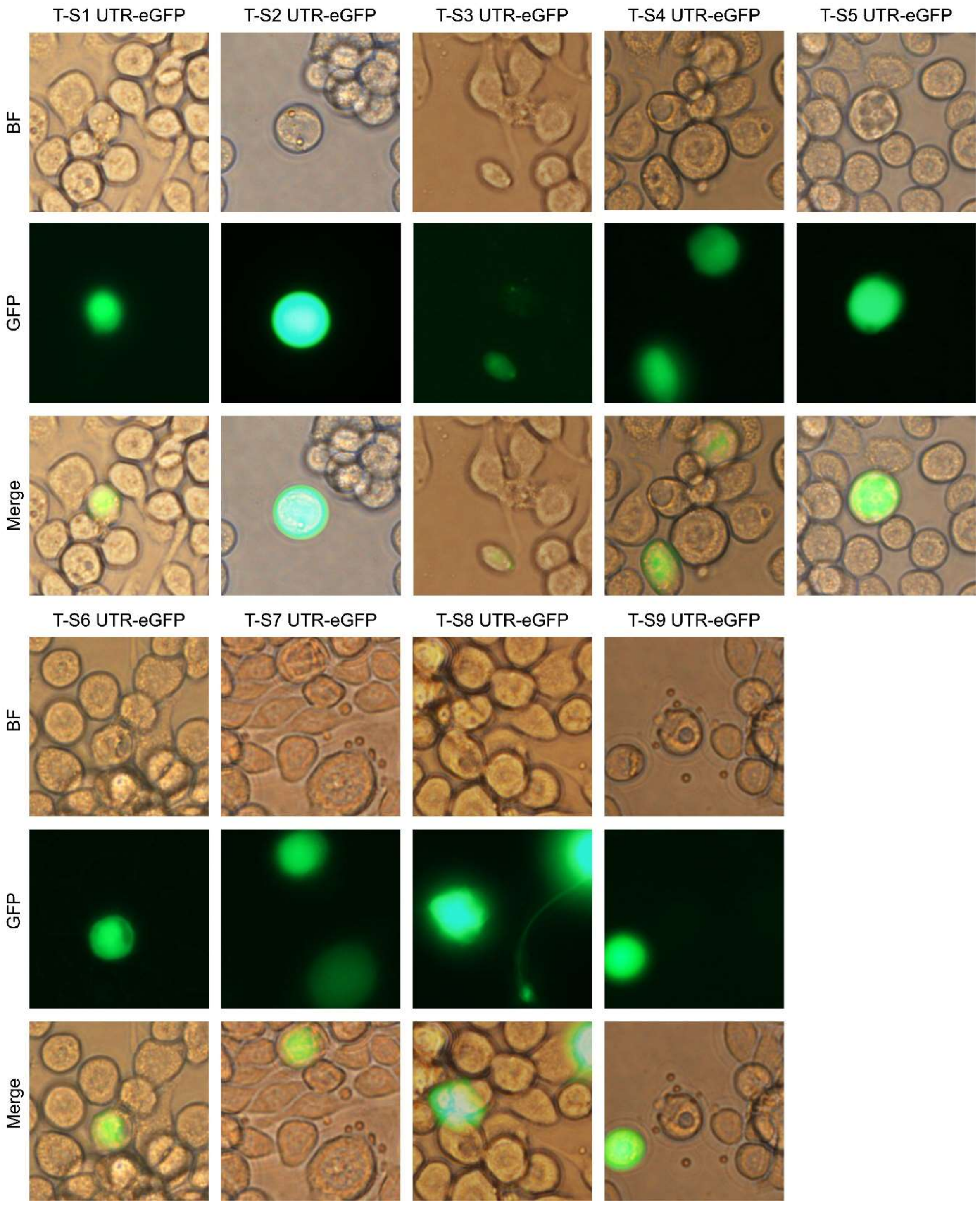

Figure S3. Sf9 cells were transfected with the three DpCPV constructs (pBS-S10-S7-S1-S9M, pBS-S6-S5-S3 and pBS-S8-S4-S2), AcBac-T7pol-Δvp80 and one of the additional vectors, T-S1UTR-egfp to T-S9UTR-egfp. Images were obtained by fluorescence microscopy with 488-nm light excitation at 7 days post-transfection. OBs and green fluorescence could be observed in the same cell only when the ORF region of the S2 gene was replaced with the *egfp* gene. In the ot her groups, no co-localization of green fluorescence and OBs was observed.
